# Racial and Ethnic Differences in Rates and Age of Diagnosis of Autism Spectrum Disorder

**DOI:** 10.1001/jamanetworkopen.2022.39604

**Published:** 2022-10-31

**Authors:** Hoangmai H. Pham, Neil Sandberg, Jeff Trinkl, Johnston Thayer

**Affiliations:** 1 Institute for Exceptional Care, Washington, DC; 2 Epic Systems Corporation, Madison, Wisconsin

## Abstract

This cohort study uses electronic health record data to assess racial and ethnic disparities in prevalence or median age of diagnosis of autism spectrum disorder in children.

## Introduction

Early diagnosis of autism spectrum disorder (ASD) is critical for ensuring that children receive appropriate and timely clinical, behavioral, and social support and treatment.^1^ Research has reported racial and ethnic disparities, with White children diagnosed earlier and more often than children in other racial and ethnic groups,^2^ and negative outcomes resulting from these disparities.^3^ A 2019 report from the Centers for Disease Control and Prevention (CDC) suggests these gaps are closing.^4^ This cohort study assesses racial and ethnic parity across rates and age at ASD diagnosis.

## Methods

To assess racial and ethnic disparities in prevalence or median age of ASD diagnosis, data were reviewed from Cosmos,^5^ a Health Insurance Portability and Accountability Act–defined limited data set using deidentified electronic health record (EHR) data from more than 140 million patients across 50 states, served by 163 health care organizations representing 960 hospitals and more than 20 000 clinics. Information on ASD diagnosis and race and ethnicity classifications is available in eMethods in the Supplement. This study was exempt from institutional review board approval and the need for informed consent in accordance with 45 CFR §46.102. This report follows the STROBE reporting guideline for cohort studies.

Consistent with a previous report from the CDC,^6^ we limited the study population to patients aged 8 years on the last date for each calendar year to control for population distributions of age by race. Patients had to have at least 1 visit per calendar year from 2017 to 2021 to exclude those who may have stopped seeking care or changed clinicians. We computed prevalence ratios in the study population for each year from 2017 to 2021.

We assessed median age at diagnosis across racial and ethnic groups by examining EHR data for patients aged 8 years with an observed ASD diagnosis in 2021 to determine when they were first diagnosed with ASD. Analyses were conducted using the Python programming language version 3.9.9 (Python Software Foundation) and SQL Server Management Studio, version 18.5.1 (Microsoft).

## Results

The study population included 1 489 594 patients (4% Asian or Other Pacific Islander; 18% Black; 21% Hispanic; and 56% White). In 2017, the prevalence rate of ASD diagnosis in Black children was 2.05% (95% CI, 1.93-2.17) compared with 2.30% (95% CI, 2.23-2.38) for White children. However, in 2021, the prevalence rate in Black children was 4.01% (95% CI, 3.85-4.17) compared with 3.89% (95% CI, 3.80-3.98) in White children (Table).

**Table.** Prevalence of ASD Diagnosis Across Racial and Ethnic Groups

Year	Race and ethnicity^a^											
	Asian or Other Pacific Islander			Black			Hispanic			White (non-Hispanic)		
	Total No.	ASD diagnosis found, No.	% (95% CI)	Total No.	ASD diagnosis found, No.	% (95% CI)	Total No.	ASD diagnosis found, No.	% (95% CI)	Total No.	ASD diagnosis found, No.	% (95% CI)
2017	12 095	260	2.15 (1.91-2.42)	52 546	1076	2.05 (1.93-2.17)	63 029	1311	2.08 (1.97-2.19)	168 614	3886	2.30 (2.23-2.38)
2018	11 928	310	2.60 (2.33-2.90)	51 532	1495	2.90 (2.76-3.05)	60 922	1618	2.66 (2.53-2.79)	163 311	4723	2.89 (2.81-2.97)
2019	12 592	396	3.14 (2.85-3.46)	52 184	1793	3.44 (3.28-3.60)	61 848	2099	3.39 (3.25-3.54)	164 961	5674	3.44 (3.35-3.53)
2020	14 144	533	3.77 (3.47-4.09)	54 591	2045	3.75 (3.59-3.91)	63 764	2485	3.90 (3.75-4.05)	169 454	6282	3.71 (3.62-3.80)
2021	14 131	632	4.47 (4.14-4.83)	57 995	2324	4.01 (3.85-4.17)	65 596	2781	4.24 (4.09-4.40)	174 357	6778	3.89 (3.80-3.98)

Abbreviation: ASD, autism spectrum disorder.

^a^ Race and ethnicity were defined based on data entered into the electronic health record. Because the software allows recording of multiple races and ethnicities per patient, the first race recorded was used.

We found that age of diagnosis was consistent across racial and ethnic groups adjusted for age differences across the underlying population. At the end of the study period, the median age at diagnosis in all groups was between 4.4 and 4.9 years, with the youngest mean age at diagnosis of 4.49 years (95% CI, 4.38 to 4.61 years) in Asian children and the oldest mean age of 4.97 years (95% CI, 4.94 to 5.02 years) in White children. The estimate for Black children was 4.89 years (95% CI, 4.83 to 4.96 years), and for Hispanic children was 4.77 years (95% CI, 4.71 to 4.83 years).

## Discussion

Addressing racial and ethnic disparities in ASD diagnosis is critical for improving outcomes for children in racial and ethnic minority populations. We found that racial and ethnic disparities in ASD diagnosis changed from 2017 to 2021. This change might be due to more effective outreach to minority communities and efforts to improve ASD screening overall.^6^

One study limitation is that EHR data for date of diagnosis might not reflect the time of diagnosis if a different clinician made the diagnosis. Our results may not be generalizable to the national population to the extent that clinicians serving disproportionate numbers of minority patients did not participate in Cosmos or minority patients were less likely to seek care. In addition, data quality for events during the study period are likely better than historical information, making incidence estimates potentially more accurate for younger patients.

## Notes

Supplement. **eMethods.** Data Definitions and Definitions of Race and Ethnicity

## References

[1] Hosozawa M, Sacker A, Cable N. Timing of diagnosis, depression and self-harm in adolescents with autism spectrum disorder. Autism. 2021;25(1):70-78. doi:10.1177/1362361320945540 32772703 PMC8162135

[2] Mandell DS, Listerud J, Levy SE, Pinto-Martin JA. Race differences in the age at diagnosis among Medicaid-eligible children with autism. J Am Acad Child Adolesc Psychiatry. 2002;41(12):1447-1453. doi:10.1097/00004583-200212000-00016 12447031

[3] Aylward BS, Gal-Szabo DE, Taraman S. Racial, ethnic, and sociodemographic disparities in diagnosis of children with autism spectrum disorder. J Dev Behav Pediatr. 2021;42(8):682-689. doi:10.1097/DBP.0000000000000996 34510108 PMC8500365

[4] The Centers for Disease Control and Prevention. Spotlight on: racial and ethnic differences in children identified with autism spectrum disorder (ASD). Accessed on February 4, 2022. https://www.cdc.gov/ncbddd/autism/addm-community-report/differences-in-children.html

[5] Tarabichi Y, Frees A, Honeywell S, ; The Cosmos Collaborative. A vendor-facilitated electronic health record data aggregation platform. ACI open. 2021;5(1):e36-e46. doi:10.1055/s-0041-1731004 35071993 PMC8775787

[6] The Centers for Disease Control and Prevention. Autism spectrum disorder: 2021 community report: data for action. Accessed on March 7, 2022. https://www.cdc.gov/ncbddd/autism/addm-community-report/data-for-action.html

