# Supplementary material for: Racial and Ethnic Differences in Rates and Age of Diagnosis of Autism Spectrum Disorder
Source: JAMA Netw Open. 2022 Oct 31;5(10):e2239604. doi: 10.1001/jamanetworkopen.2022.39604 (PMC9623438; doi:10.1001/jamanetworkopen.2022.39604)
Supplement: Supplement. — eMethods. Data Definitions and Definitions of Race and Ethnicity [file jamanetwopen-e2239604-s001.pdf]

Supplemental Online Content

Pham HH, Sandberg N, Trinkl J, Thayer J. Racial and ethnic differences in rates and age of diagnosis of autism spectrum disorder. *JAMA Netw Open.* 2022;5(10):e2239604. doi:10.1001/jamanetworkopen.2022.39604

eMethods. Data Definitions and Definitions of Race and Ethnicity

This supplemental material has been provided by the authors to give readers additional information about their work.

© 2022 Pham HH et al. *JAMA Network Open*.

eMethods

Data Definitions

Term	Definition
**Autism Spectrum Disorder**	ICD-10-CM codes F84.0, F84.1, F84.2, F84.3, F84.4, F84.5, F84.8, F84.9
**Study Period**	1/1/2017 to 12/31/2021

Definitions of Race and Ethnicity

Race and ethnicity were defined based on data entered into the electronic health record. Because EPIC allows recording of multiple races/ethnicities per patient, we used the first race recorded. We categorized non-Hispanic whites as “white,” and similarly so for non-Hispanics in other race categories.

© 2022 Pham HH et al. *JAMA Network Open*.
